# Immunogold scanning electron microscopy can reveal the polysaccharide architecture of xylem cell walls

**DOI:** 10.1093/jxb/erx103

**Published:** 2017-04-08

**Authors:** Qiang Sun, Yuliang Sun, Kevin Juzenas

**Affiliations:** 1 Department of Biology, University of Wisconsin, Stevens Point, WI 54481, USA; 2 School of Medicine, Boston University, Boston, MA 02118, USA

**Keywords:** Cell wall, cell wall monoclonal antibody (mAb), grapevine, hemicellulose, immunofluorescence microscopy (IFM), immunogold SEM, immunogold TEM, pectin, polysaccharide architecture, xylem.

## Abstract

Immunofluorescence microscopy (IFM) and immunogold transmission electron microscopy (TEM) are the two main techniques commonly used to detect polysaccharides in plant cell walls. Both are important in localizing cell wall polysaccharides, but both have major limitations, such as low resolution in IFM and restricted sample size for immunogold TEM. In this study, we have developed a robust technique that combines immunocytochemistry with scanning electron microscopy (SEM) to study cell wall polysaccharide architecture in xylem cells at high resolution over large areas of sample. Using multiple cell wall monoclonal antibodies (mAbs), this immunogold SEM technique reliably localized groups of hemicellulosic and pectic polysaccharides in the cell walls of five different xylem structures (vessel elements, fibers, axial and ray parenchyma cells, and tyloses). This demonstrates its important advantages over the other two methods for studying cell wall polysaccharide composition and distribution in these structures. In addition, it can show the three-dimensional distribution of a polysaccharide group in the vessel lateral wall and the polysaccharide components in the cell wall of developing tyloses. This technique, therefore, should be valuable for understanding the cell wall polysaccharide composition, architecture and functions of diverse cell types.

## Highlight

A versatile immunogold scanning electron microscopy technique helps reveal the polysaccharide composition and architecture of cell walls of xylem elements.

## Introduction

Understanding cell wall polysaccharide architecture (i.e. polysaccharide components and their spatial organization in a cell wall) is essential to reveal the structure and function of the cell wall and even of the cell itself (Willats *et al.*, 2001*b*; Knox, 2008; Albersheim *et al.*, 2010; Cosgrove, 2014). In the past two decades, two major types of molecular probes have been used to detect polysaccharides in cell walls, namely monoclonal antibodies (mAbs; Puhlmann *et al.*, 1994; Knox, 1997; Willats *et al.*, 2002) and non-catalytic carbohydrate-binding modules (CBMs; Hachem *et al.*, 2000; Boraston *et al.*, 2004; McCartney *et al.*, 2004). Most mAbs are generated in either rat or mouse using specific cell wall polysaccharides as immunogens (Willats *et al.*, 2000). CBMs are recombinant his-tagged proteins derived from the specific, non-glycoside hydrolases of microorganisms and/or plants (e.g. Hachem *et al.*, 2000; Szabo *et al.*, 2001; McCartney *et al.*, 2006). Their steric specificity to individual polysaccharides or polysaccharide groups makes them valuable tools for detecting cell wall polysaccharides *in situ*. As the number of the cell wall mAbs and CBMs has expanded, so have the possibilities of detecting more cell wall polysaccharides (Pattathil *et al.*, 2010; Hervé *et al.*, 2011; Johnsen *et al.*, 2015).

In order to detect cell wall polysaccharides *in situ*, a specially tagged secondary Ab is used to specifically bind to the cell wall probe at the epitope(s) of target polysaccharides. Since the tag of the secondary Ab provides a visible signal in the light or electron microscope with certain settings, a polysaccharide or polysaccharide group can be localized in a specimen of plant tissue. The signal strength from the secondary antibody tag may also be used for relative quantification of the polysaccharide concentrations. Currently, an immunohistochemical/immunocytochemical protocol using the molecular probes and certain fluorophore-conjugated secondary Abs along with fluorescence microscopy (a.k.a., immunofluorescence microscopy; IFM) has become the most common technique to localize cell wall polysaccharides at the tissue or cellular level. This technique has been used extensively to explore cell wall polysaccharides and their functional roles (Orfila and Knox, 2000; Willats *et al.*, 2002; Sun *et al.*, 2011; Cosgrove, 2014). Another technique combining the molecular probes, colloidal gold-conjugated secondary Abs and transmission electron microscopy (TEM) (a.k.a., immunogold TEM) was also established to immunolocalize polysaccharides across the thickness of a cell wall at much higher resolution. The immunogold TEM can also provide information on the relative quantities of target polysaccharides in a cell wall (Liners and Van Custem, 1992; Rioux *et al.*, 1998; Herbette *et al.*, 2015). Therefore, both methods can be beneficial for analysing the composition and distribution of polysaccharides depending on the questions being investigated.

Despite their important role in localizing polysaccharides, both IFM and immunogold TEM have been used to detect polysaccharides mostly in transverse sections of cell walls. Because of limitations either in resolution of IFM or in sample size of immunogold TEM, neither provides adequate three-dimensional information to resolve the polysaccharide architecture of a cell wall. To better understand the spatial distribution of polysaccharides in a cell wall, the ability to visualize the distribution of polysaccharides at high resolution over a large cell wall surface becomes crucial. With its high resolution, large field of view, and stereoscopic capabilities, scanning electron microscopy (SEM) should be an excellent tool for studying a cell wall’s surface structure. When combined with cell wall mAbs and colloidal gold-conjugated secondary Abs, the immunogold SEM should provide some major advantages over IFM and immunogold TEM in visualizing target molecules over a large surface area. Furthermore, normally concealed underlayers of the thick cell walls of many cell types can be exposed after the more superficial wall layers are removed during the preparation of SEM specimens (Sun *et al.*, 2013). Immunolocalizing polysaccharides on the exposed underlayers with the immunogold SEM should provide valuable information for reconstructing the three-dimensional distribution of polysaccharides in the cell walls. Therefore, immunogold SEM can be an ideal tool for analysing the polysaccharide architecture of a cell wall. Immunogold SEM protocols have been developed for a variety of mammalian tissues, cells and organelles to detect proteins and other molecules on their structural surfaces (Lilje and Armati, 1995; Hermann *et al.*, 1996; Goldberg and Fiserova, 2010). While such a method has been used to detect the distribution of proteoglycans on the surface of root and callus of maize (Samaj *et al.*, 1999), there is still a need for a robust immunogold SEM protocol that can be applied to study the cell wall polysaccharide architectures of diverse cell types, xylem cells in particular.

In recent years, we developed an effective immunocytochemical protocol with confocal laser scanning microscopy to visualize multiple groups of polysaccharides in the cell walls of xylem elements (Sun *et al.*, 2011). Based on that protocol, we have used grapevines as a model in this study to explore an optimal immunogold SEM protocol that can effectively detect pectic and hemicellulosic polysaccharides in the cell walls of different types of xylem cells.

Vessel elements are an important cell type in xylem. Multiple vessel elements are connected end to end to form individual vessels in a xylem tissue, which are involved in water conduction, structural support and, in the disease state, vascular occlusion (Esau, 1977; Tyree and Zimmermann, 2002). It is well known that the diversity of vessel functionality is closely related to the structural differentiation in the lateral wall of its vessel elements, that is, wall regions with a thick secondary cell wall and those containing intervessel and vessel-parenchyma pit pairs (PPs). Understanding the polysaccharide composition and distribution in these structurally differentiated wall regions will help clarify their functional roles. The current study tested how the immunogold SEM technique may be used to analyse the spatial distribution and quantity of certain pectic and hemicellulosic polysaccharides in the lateral wall of vessel elements.

Tylose is a common cellular structure derived from a parenchyma cell, which develops into a vessel lumen in many species in response to diverse stress conditions, such as wounding and pathogen infection (Esau, 1977; Evert, 2006). Its development in xylem may result in vascular occlusion, thereby helping wound sealing or contributing to disease resistance/susceptibility of a host plant (Carlquist, 2001; Sun *et al.*, 2006, 2011). Cell wall expansion is a key aspect of tylose development. In this study, we have tested the applicability of our immunogold SEM protocol to reveal cell wall pectic and hemicellulosic polysaccharides of developing tyloses. All of these analyses will help to clarify polysaccharide composition and organization, consequently contributing to a better understanding of the structure and function of cell walls.

## Materials and methods

### Plant materials and experimental treatments

The materials for this investigation included five grapevine varieties/genotypes: *Vitis vinifera* var. Chardonnay, *V. arizonica* var. B43-17, *V. vinifera* × *arizonica* vars. U0505-35 and 0505-01, and *V. vinifera* × *rupestris* var. 89–0908. Six vines of each genotype were grown in a 7.6 l pot with a 16 h light/8 h dark daily cycle in the Biology Department Greenhouse at the University of Wisconsin–Stevens Point and were trained to retain two shoots, with each growing from a robust bud at the common short scion trunk. Each shoot was maintained at a total of 20–25 internodes in height by pruning off the top and regularly pruning off some lateral branches.

When each shoot was 12–14 weeks old, a 3 cm-long internode length was collected from the upper portion of the 10th internode, counting from the shoot base. To induce tylose development, the remaining end of each shoot was kept exposed to air for one more week (Sun *et al.*, 2006, 2008). A second 3 cm-long length of stem including the exposed end was obtained from each shoot for tylose analysis. All the samples collected from each vine were immediately fixed in 4% paraformaldehyde in PEM buffer (50 mM PIPES, 5 mM EGTA, 5 mM MgSO_4_, pH 6.9; Willats *et al.*, 2002) for at least 24 h before examinations.

### Light microscopy and conventional SEM for xylem structure analyses

Both light microscopy and conventional (thermal-emission) SEM were used to study xylem structural features of the five grapevine genotypes. For light microscopy, each pre-fixed internode length was washed in 50 mM PIPES twice for 10 min each. Some 1 mm-thick transverse, radial and tangential segments (with a surface dimension of 3 mm × 3 mm) of secondary xylem were cut, respectively, from each pre-fixed sample in the same buffer. Segments were washed twice in the buffer, 30 min each, and then dehydrated in 20-min steps from 10% to 90% ethanol in 10% steps and two changes of 95%. After that, xylem segments were infiltrated on a rotator with a 50% mixture of L. R. White resin (hard grade, precatalysed) and 95% ethanol and two changes of 100% resin, with the first two steps for 1 h each and the last step for 12 h. Each resin-infiltrated xylem segment was transferred to a mold filled with fresh 100% L. R. White resin and cured for 24 h under UV light at about –20 °C in a UV cryo chamber (Pelco UVC3, Ted Pella, Inc., USA). For light microscopy, resin-embedded xylem tissue was thick-sectioned (*ca* 1 µm) with a glass knife on an ultramicrotome (Leica EM UC6, Leica Microsystems GmbH, Austria). Sections were stained with 0.5% toluidine blue in 0.5% sodium borate, examined with a compound light microscope (Nikon Eclipse 50i, Nikon Corp., Japan) and photographed with a digital camera (Nikon Digital Sight-5Mc, Nikon Corp., Japan).

Conventional SEM was used to study xylem structural features by following the procedures described in Sun *et al.* (2013). In brief, xylem segments were cut from each pre-fixed internode length and then dehydrated in ethanols as described above with the addition of two 30-min changes of 100% ethanol. Dehydrated specimens were critical-point-dried (DCP-1, Denton Vacuum, Inc., USA), sputter-coated with gold-palladium (Desk II, Denton Vacuum, Inc., USA) and examined under a scanning electron microscope (Hitachi S3400N, Hitachi Science Systems, Ltd, Japan) with the secondary electron detector at an accelerating voltage of 5 or 8 kV.

### Immunogold labeling and its negative controls of xylem tissue

Four cell wall mAbs, JIM5, JIM7, CCRC-M1 and CCRC-M140, were used as the primary Abs to detect certain pectic and hemicellulosic polysaccharides in the cell walls of secondary xylem elements. JIM5 and JIM7 are two rat-derived Abs from the PlantProbes (University of Leeds, UK) that bind specific epitopes of homogalacturonans (HGs), recognizing weakly methyl-esterified HGs (Me-HGs) and heavily Me-HGs, respectively (VandenBosch *et al.*, 1989; Knox *et al.*, 1990; Willats *et al.*, 2000; Clausen *et al.*, 2003). CCRC-M1 and CCRC-M140 are mouse-derived Abs from the CarboSource Service (University of Georgia, USA), recognizing two major groups of hemicellulosic polysaccharides: fucosylated xyloglucans (XyGs) and xylans, respectively (Puhlmann *et al.*, 1994; Freshour *et al.*, 2003; Pattathil *et al.*, 2010). Two kinds of 10 nm colloidal gold-conjugated secondary Abs, goat anti-rat IgG (whole molecule, Sigma-Aldrich batch no. 129K2049) and goat anti-mouse IgG (whole molecule, Sigma-Aldrich batch no. 089K0961), were used to localize the JIM and CCRC-M mAbs, respectively.

Xylem segments for immunogold SEM were obtained from each pre-fixed internode as described above for light microscopy. Segments were washed in 50 mM PIPES twice for 30 min each and blocked with 3% non-fat milk powder in 0.2 M phosphate-buffered saline (MP/PBS, pH 7.4) at room temperature (RT) for 1 h. Then, xylem segments were divided into four sets: one for immunogold labeling with one cell wall mAb and its corresponding secondary Ab and the other three for three types of negative controls of the immunogold labeling, respectively. For immunogold labeling, specimens were incubated overnight in a cell wall mAb solution at 4 °C, followed by three PBS washes, 10 min each. They were then transferred to the corresponding colloidal gold-conjugated secondary Ab solution for 1 h at RT. Specimens were washed twice with PBS and twice with distilled deionized water (DD H_2_O), 10 min each change. Because the 10 nm colloidal gold particle is below the conventional SEM’s resolution, the gold particle was silver-enhanced as follows: washed specimens were silver-enhanced at RT in a darkroom by submerging them in a droplet of freshly made initiator–developer mix (1:1) from a silver enhancing kit (BBI Solutions, UK, batch no. 12348). This treatment deposits a silver layer on the gold particles conjugated to secondary Ab, forming larger silver-enhanced gold particles that appear as bright particles resolvable under the SEM at a certain accelerating voltage with either the secondary electron (SE) or the backscattered electron (BSE) detector. Therefore, the bright particles indicate the presence and location of the polysaccharide or polysaccharide group detected by a cell wall Ab. Details of the concentration and incubation time of both primary and secondary Abs and the time for silver enhancement treatment are described below.

In order to demonstrate the effectiveness and reliability of the xylem immunogold labeling treatment, three kinds of negative controls were run for each cell wall mAb. Specimens were prepared and processed exactly as described above except that the mAb, its corresponding secondary Ab, and both the mAb and the secondary Ab were replaced with 3% MP/PBS, respectively, in the three controls.

Our previous study indicated that the optimal concentrations of both primary and secondary Abs are important to obtain the strongest signals from target molecules with a minimal level of background noise caused by non-specific Ab binding (Sun *et al.*, 2011). Trials with combinations of different concentrations of each cell wall mAb and its corresponding secondary Ab and different times of silver enhancement treatment were first conducted with *V. vinifera* var. Chardonnay to explore the optimal conditions for the best signal/noise ratio. The concentrations tested included undiluted, 3-, 10-, 30-, and 100-fold dilutions of each mAb’s hybridoma supernatant in 3% MP/PBS and undiluted, 25-, 50-, 100-, and 200-fold dilutions of each secondary Ab also in 3% MP/PBS. The time for the silver enhancement treatment was tested at 5, 10, 15, 20, and 25 min. Based on the trials, the optimal combination of the concentrations of each mAb and its corresponding secondary Ab and the time for the silver enhancement treatment were determined and used to visualize cell wall polysaccharides in all of the other specimens from the grapevine genotypes used in the study. For each mAb (either the immunogold labeling or each of the three negative controls), five to ten samples from each genotype were used for cell wall polysaccharide detection.

### Visualization of pectic and hemicellulosic polysaccharides in cell walls with SEM

Silver-enhanced specimens were washed in DD H_2_O three times with 10 min each, dehydrated, critical-point-dried and sputter-coated with gold-palladium under the conditions previously described. Coated specimens were then observed under the same SEM. Both accelerating voltage and detection mode of an SEM may affect how well the silver-enhanced gold particles can be distinguished from their background cell wall structure. The accelerating voltage was first tested at 3, 5, 8, and 10 kV, respectively, with either the SE or BSE detector by using specimens of *V. vinifera* var. Chardonnay. The optimal SEM conditions were then determined and used for all other immunogold-labeled specimens and their negative controls.

## Results

### Xylem elements and their cell wall structural features

**Fig. 1.**
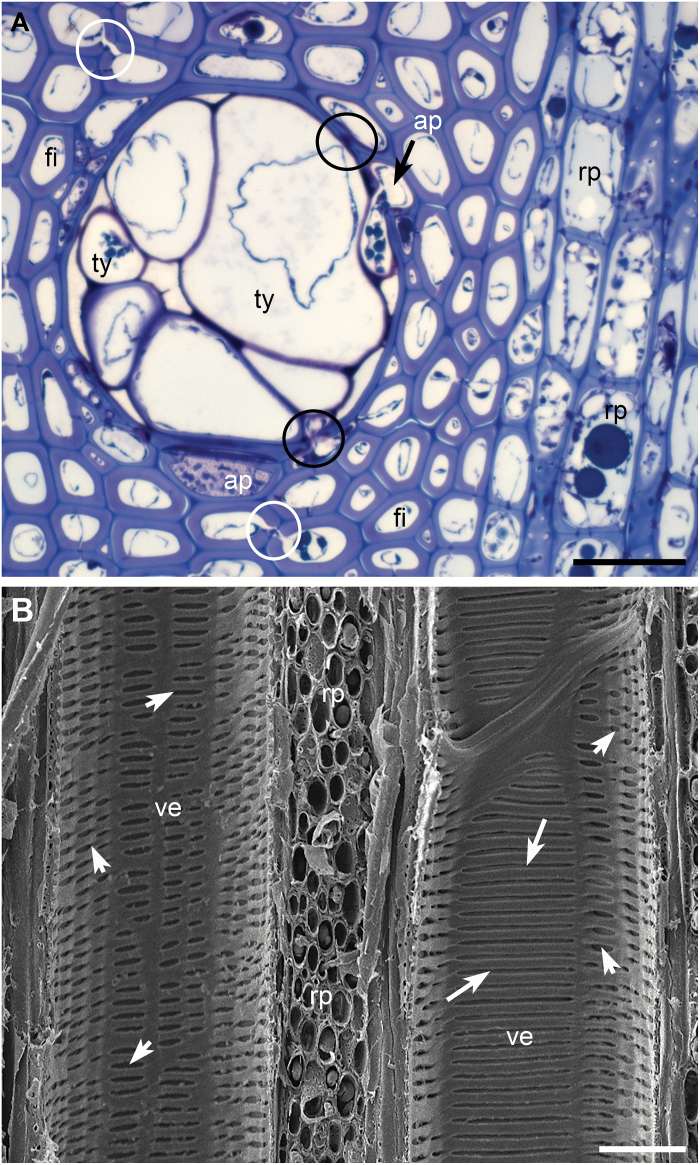
Arrangements of secondary xylem elements in stems of *Vitis vinifera* var. Chardonnay. (A) Xylem tissue viewed in the transverse section under a light microscope, showing a tylose (ty)-filling vessel, axial parenchyma cells (ap), fibers (fi), ray parenchyma cells (rp), vessel-parenchyma PPs (black circles) and interfiber PPs (white circles). (B) An SEM image of xylem tissue in a tangential longitudinal section, showing two transected vessels (ve) sandwiching ray parenchyma cells (rp). The vessel on the left has relatively smaller, horizontally elongated pits (short arrows) of vessel-parenchyma PPs in its lateral wall. The vessel on the right contains pits of both vessel-parenchyma PPs (short arrows) and scalariformly arranged intervessel PPs (long arrows) in its lateral wall. Bar in each panel: 40 µm.

Secondary xylem of grape stems contained four main types of cells: vessel elements, fiber cells, axial parenchyma cells and ray parenchyma cells (Fig. 1A). In the transverse section, a vessel element may have a direct wall contact with up to all the four types of cells (Fig. 1A). Pit pairs (PPs) occurred where wall regions made contact, except where a fiber cell contacted a vessel element. The PPs between a vessel element and an axial or ray parenchyma cell (vessel-parenchyma PPs, Fig. 1A) were half-bordered with a bordered pit on the vessel side and a simple pit on the parenchyma cell side. When viewed from the vessel side, pits were round, oval, or horizontally elongated (Fig. 1B and see Fig. 3A, C). When a stem was wounded, some parenchyma cells surrounding a vessel grew through their vessel-parenchyma PPs to form tyloses in the lumen of the vessel, partially or completely blocking the lumen (Fig. 1A and see Fig. 7A). Bordered PPs occurred between adjacent vessel elements (intervessel PPs) and were scalariformly arranged with each PP extending horizontally the full contact wall width of the adjacent vessels (Fig. 1B). Secondary wall borders of an intervessel pit were arched over the intervessel pit membrane (PM), allowing only a small central stripe area of the PM to be visible from the surface view of vessel lateral wall. The whole intervessel PM surface became viewable only when the secondary wall borders were removed during the sample trimming process (see Figs 3B and 4A). Adjacent fiber cells had few simple PPs (Fig. 1A and see Fig. 5B, C). Adjacent axial or ray parenchyma cells had round or oval bordered PPs with narrow secondary wall borders (see Fig. 6A, B). Other general structural features of secondary xylem and intervessel and vessel-parenchyma PPs in grapevine stems have been described in Sun *et al.* (2006, 2011).

### Optimal SEM settings for immunolocalizing polysaccharides under an SEM

**Fig. 2.**
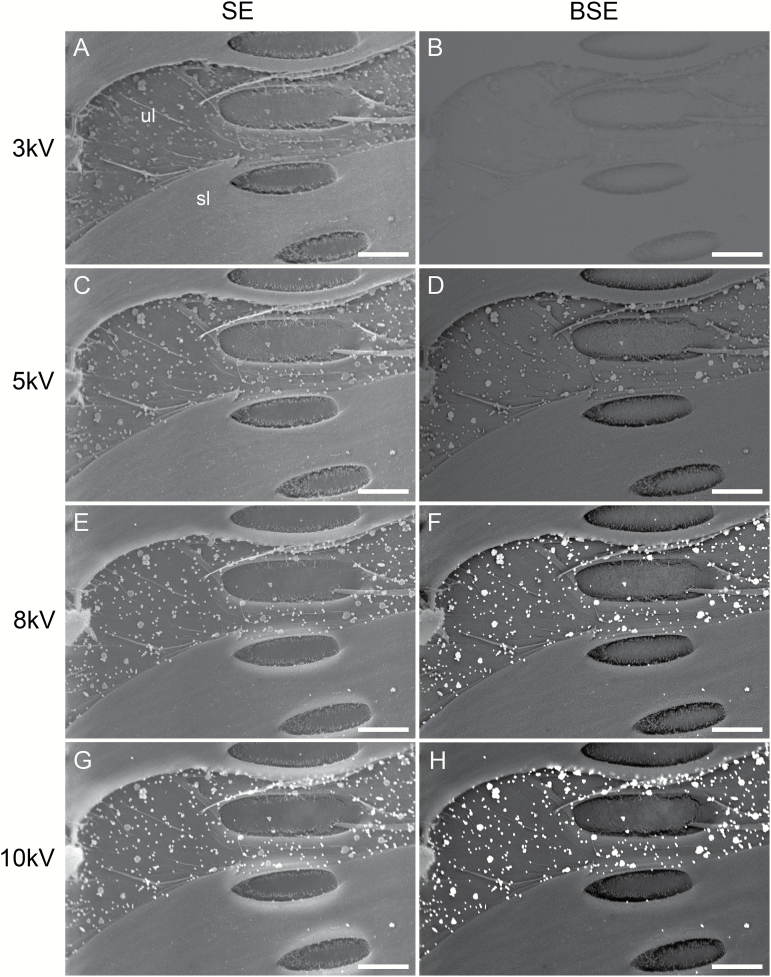
Optimization of SEM accelerating voltage and electron detection mode for detecting cell wall polysaccharides in xylem tissue. Shown are representative images of a vessel lateral wall in secondary xylem of *V. vinifera* var. Chardonnay after incubation with CCRC-M140 and the colloidal gold-conjugated goat anti-mouse IgG followed by silver enhancement. Part of the lateral wall has exposed its originally concealed secondary wall underlayers (ul) after removal of a superficial secondary wall layer (sl). Bright particles on the underlayers are silver-enhanced gold particles, indicating the presence of the polysaccharides (xylans) recognized by the cell wall mAb. All images were taken at the same magnification and location but under different combinations of four accelerating voltages (3 kV: A, B; 5 kV: C, D; 8 kV: E, F; and 10 kV: G, H) and two electron detection modes (SE mode: A, C, E, G; and BSE mode: B, D, F, H). (A, B) Silver-enhanced particles are hardly distinguishable (A) and not distinguishable at all (B) from the background wall structures in terms of brightness. (C, D) Bright silver-enhanced particles are more or less distinguishable from the background wall structures with better structural details/resolution shown under the SE mode (C) but higher contrast, bright silver-enhanced particles standing out in the BSE mode (D). (E–H) Bright particles of different sizes are readily distinguishable and the background wall structural details are better revealed under the SE mode (E, G) than the BSE mode (F, H). Bar in each panel: 5 µm.

Under optional conditions, silver-enhanced gold particles appeared as bright particles under SEM (Fig. 2). The ability to distinguish silver-enhanced gold particles from background on our specimens depended largely on the energy level of the initial electron beam and the electron detection mode. The former is proportional to the accelerating voltage for the initial electron beam, while the latter detects signals from either secondary electrons (SE mode) or backscattered electrons (BSE mode). The SE detection mode was better at revealing structural details and the mass-sensitive BSE detection mode at detecting heavy metal elements under a thin coating. At 3 kV of the accelerating voltage, silver-enhanced gold particles remained more or less indistinguishable in the SE mode (Fig. 2A) or not distinguishable at all in the BSE mode (Fig. 2B) from other background structures. At 5 kV, silver-enhanced particles were more or less distinguishable from background structures with the SE mode (Fig. 2C) but appeared as bright particles from a relatively dark background with the BSE mode (Fig. 2D). At 8 kV and greater, bright silver-enhanced particles were readily distinguished from the background even at low magnifications in either the SE or BSE mode (Fig. 2E–H), although better contrast images with much brighter particles were observed with the BSE mode (Fig. 2F, H). Accelerating voltages above 8 kV did not markedly improve resolution and detection of silver-enhanced particles but sometimes showed noticeable damage to delicate structures like PMs. On the other hand, the BSE mode was found to have reduced resolution for structural analyses compared with the SE mode. With all of these considered, the optimal SEM settings for immunolocalizing cell wall polysaccharides used in this study were determined to be an accelerating voltage of 8 kV with either the SE or BSE detection mode, or an accelerating voltage of 5 kV with the BSE detection mode.

### Optimal combinations of Ab concentrations and silver enhancement treatment time in detecting cell wall polysaccharides

**Fig. 3.**
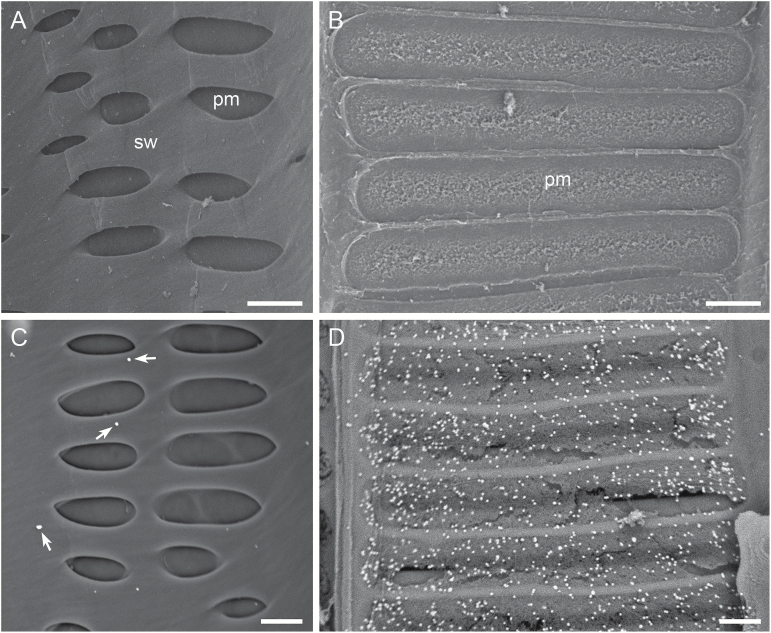
Reliability and effectiveness of the immunogold SEM technique in detecting pectic and cellulosic polysaccharides in the cell wall of xylem elements. Shown are representative images of the lateral wall of vessel elements from *V. vinifera* var. Chardonnay in the three negative controls (A–C) and one immunogold labeling with CCRC-M1 and the colloidal gold-conjugated goat anti-mouse IgG (D), both of which were followed by the silver enhancement treatment. Images were taken in the SE mode at an accelerating voltage of 8 kV. The bright silver-enhanced gold particles indicate the presence of fucosylated XyGs. (A) A vessel lateral wall with vessel-parenchyma pits and PMs (pm), and other wall regions containing the intact secondary wall (sw). The tissue was not incubated with the mAb and the secondary Ab and bright particles are absent on the vessel lateral wall. (B) A vessel lateral wall with multiple intervessel PMs (pm) visible after removal of the overarching secondary wall borders. The xylem tissue was incubated with the mAb but without the secondary Ab, and no bright particles are visible on the PMs. (C) A vessel lateral wall with vessel-parenchyma pits in a xylem tissue incubated without the mAb but with the secondary Ab has very few randomly distributed bright particles (arrows). (D) Intervessel PMs in a xylem tissue incubated with both the mAb and the secondary Ab are associated with numerous bright particles, indicating the abundant presence of fucosylated XyGs. Bar in each panel: 5 µm.

**Fig. 4.**
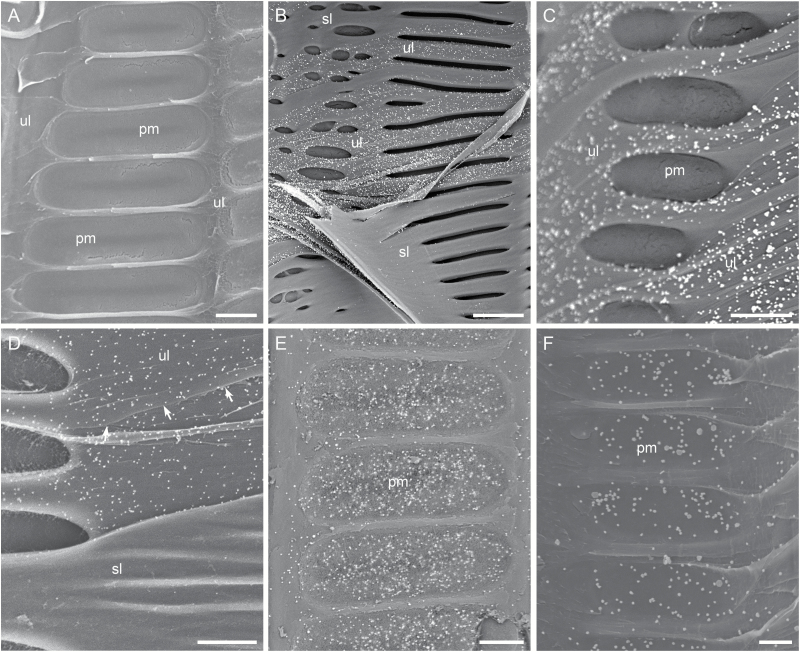
Representative images demonstrating detection of pectic and hemicellulosic polysaccharides in the lateral wall of vessel elements. Images are from *V. vinifera* var. Chardonnay (A–E) and *V. arizonica* var. B43-17 (F). Bright silver-enhanced gold particles localized the specific polysaccharide(s) recognized by a cell wall mAb. (A) Secondary xylem tissue used as a negative control. The tissue was incubated without CCRC-M140 but with the colloidal gold-conjugated goat anti-mouse IgG. Shown are multiple intervessel PMs (pm) and some regions with cell wall underlayers (ul) exposed after removal of the pit borders and superficial secondary wall layers. No bright particles are visible on the intervessel PMs and wall underlayers. (B–F) Xylem tissue incubated with CCRC-M140 (B–D), CCRC-M1 (E) or JIM5 (F) and its corresponding colloidal gold-conjugated secondary Ab. (B) A vessel lateral wall, showing that the intact secondary wall surface (sl) does not contain detectable amount of xylans but the wall underlayers (ul) have the abundant presence of the polysaccharides. (C) A vessel lateral wall with structural details showing the abundant presence of xylans in some wall underlayers (ul) but the absence in the vessel-parenchyma PMs. (D) A vessel lateral wall containing a region with the intact superficial secondary wall (sl) and a region with exposed wall underlayers (ul). The intact wall surface does not contain xylans but the exposed underlayers have xylans in the different layers (arrows indicate the location where a layer goes under another layer). (E) Intervessel PMs (pm) showing the abundant presence of fucosylated XyGs. (F) Intervessel PMs (pm) showing the presence of weakly Me-HGs. Bar equals 3 µm in (D–F), 5 µm in (A, C), and 15 µm in (B).

One key to the success of the immunogold SEM technique in detecting cell wall polysaccharides in xylem elements is the optimal combination of cell wall mAb concentration, colloidal gold-conjugated secondary Ab concentration and silver enhancement treatment time. Through the designated trials described in ‘Materials and methods’, the optimal conditions for either of the two CCRC M Abs were found to be a 3-fold dilution of the original mAb mouse hybridoma supernatant, a 50-fold dilution of the original colloidal gold-conjugated goat anti-mouse IgG and 15 min of silver enhancement treatment (Fig. 3D). The optimal conditions for either JIM5 or JIM7 were a 10-fold dilution of the mAb, a 50-fold dilution of the original colloidal gold-conjugated goat anti-rat IgG and 15 min of silver enhancement treatment (Fig. 4F). In all three types of negative controls for the immunogold labeling with each cell wall mAb, none to very few randomly distributed bright particles were observed on the surface of cell walls (Fig. 3A–C). This demonstrated that the technique developed here was reliable and effective in visualizing specific pectic and hemicellulosic polysaccharides in cell walls.

### Using immunogold SEM to localize pectic and hemicellulosic polysaccharides in the cell walls of various xylem elements

**Fig. 5.**
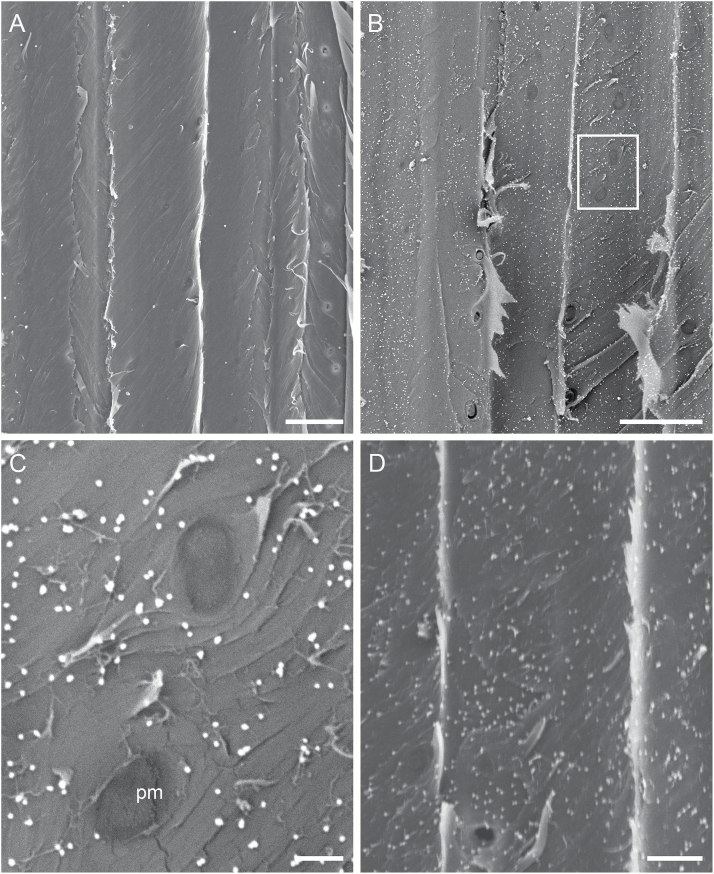
Representative images demonstrating detection of pectic and hemicellulosic polysaccharides in the lateral wall of secondary xylem fiber cells. Shown are longitudinal sectional views of fiber cell walls from *V. vinifera* var. Chardonnay mostly with secondary wall underlayers exposed after removal of some superficial secondary wall layers. Bright silver-enhanced gold particles indicate the presence of the polysaccharide(s) recognized by a specific cell wall mAb. (A) Fiber cell walls in a xylem tissue incubated without CCRC-M140 but with the colloidal gold-conjugated goat anti-mouse IgG (negative control), showing few randomly distributed bright particles. (B) Exposed secondary wall underlayers of fiber cells in a xylem tissue incubated with CCRC-M140 and the colloidal gold-conjugated goat anti-mouse IgG are visualized with numerous silver-enhanced gold particles, indicating the presence of xylans. (C) Enlargement of the frame region in (B), showing the presence of xylans in different secondary wall underlayers but not in interfiber PMs (pm). (D) Exposed secondary wall underlayers of several fiber cells in a xylem tissue incubated with CCRC-M1 and the colloidal gold-conjugated goat anti-mouse IgG are associated with silver-enhanced gold particles, indicating the presence of fucosylated XyGs. Bar equals 10 µm in (A, B), 3 µm in (D), and 1 µm in (C).

**Fig. 6.**
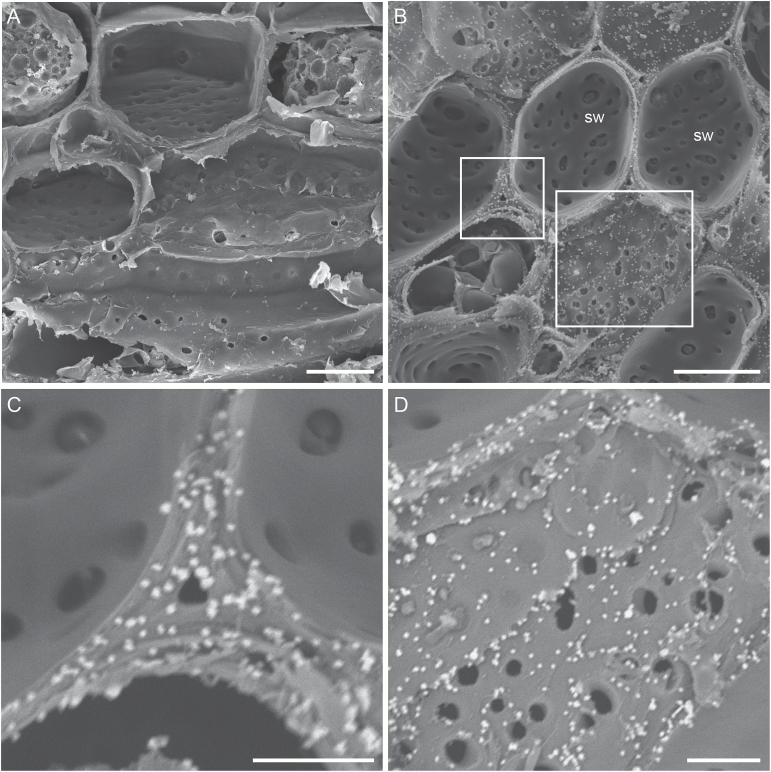
Representative images demonstrating detection of pectic and hemicellulosic polysaccharides in the cell wall of xylem parenchyma. Shown are sectional views of ray parenchyma cells with round or oval PPs in between in the secondary xylem tissue from *V. vinifera* var. Chardonnay. (A) A ray parenchyma tissue incubated without a cell wall mAb but with the colloidal gold-conjugated goat anti-mouse IgG (negative control) has very few randomly distributed bright particles on the walls of its parenchyma cells. (B–D) A ray parenchyma tissue incubated with CCRC-M1 and the colloidal gold-conjugated goat anti-mouse IgG. Bright silver-enhanced gold particles are present in certain wall regions of its parenchyma cells, indicating the presence of fucosylated XyGs in the wall regions. (B) Differential distribution of fucosylated XyGs among different ray parenchyma cell wall portions. Fucosylated XyGs are absent on the secondary wall surface (sw) of the parenchyma cells, but present on the transverse section and some underlayers of their cell walls. (C) Enlargement of the small frame region in (B) indicating the presence of fucosylated XyGs on the transverse section of ray parenchyma cell walls. (D) Enlargement of the large frame region in (B) showing that some wall underlayers of a ray parenchyma cell contain fucosylated XyGs. Bar equals 10 µm in (A, B), and 5 µm in (C, D).

**Fig. 7.**
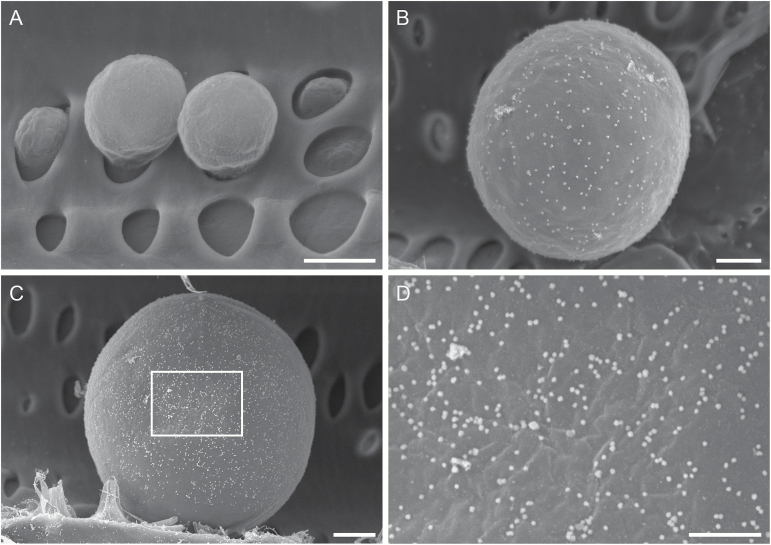
Representative images demonstrating detection of pectic and hemicellulosic polysaccharides in the cell wall of tyloses. (A) Tyloses in a xylem tissue from *V. vinifera* X *arizonica* var. U0505-35. The xylem tissue was incubated with CCRC-M1 but without the colloidal gold-conjugated goat anti-mouse Ab (negative control). No bright particles are associated with the cell walls of tyloses. (B) A tylose in a xylem tissue from *V. arizonica* var. B43-17. The tissue was incubated with JIM5 and the colloidal gold-conjugated goat anti-rat IgG. The tylose is associated with bright silver-enhanced gold particles, indicating the presence of weakly Me-HGs in its cell wall. (C, D) A tylose in xylem tissue from *V. vinifera* var. Chardonnay. The tissue was incubated with CCRC-M1 and the colloidal gold-conjugated goat anti-mouse IgG. (C) The tylose surface displays a dense distribution of bright silver-enhanced gold particles, showing the abundant presence of fucosylated XyGs on the tylose wall. (D) Enlargement of the frame region in (C) indicating a random distribution of bright particles on the wall surface of tylose. Bar equals 5 µm in (A–C) and 2 µm in (D).

Our immunogold SEM technique was used to detect two groups of pectic polysaccharides (weakly and heavily Me-HGs) and two groups of hemicellulosic polysaccharides (fucosylated XyGs and xylans) in the cell walls of vessel elements, fiber cells, axial and ray parenchyma cells, and tyloses in stem secondary xylem of the five grapevine genotypes. In all the three types of negative controls for the immunogold labeling of each mAb, no or only very few randomly distributed bright particles were observed on the lateral walls of these xylem structures (Figs 4A, 5A, 6A and 7A). This indicates that bright particles visualized on a cell wall region after the immunogold labeling with each mAb reflected the actual presence and distribution of the specific polysaccharide(s) recognized by the mAb.

A vessel lateral wall includes regions with and without pits (Fig. 1B). A wall region with pits has intervessel and/or vessel-parenchyma PMs that have only the primary cell wall exposed to the vessel lumen. The PMs may become completely visible after removal of the pits’ secondary wall borders (Fig. 4A). In a wall region without pits, removal of superficial secondary wall layers also revealed some originally concealed secondary wall underlayers and even the primary cell wall (Fig. 4B, D). The immunogold SEM technique was used to detect polysaccharides in all of these cell wall structures (Fig. 4). Bright particles were absent or rarely present in the two types of wall regions in the three negative controls of each mAb (Fig. 4A). In the specimens immunolabeled with a particular mAb, different wall structures of a vessel element displayed bright particles of different densities, suggesting quantitative differences in the specific polysaccharide or polysaccharide group among the different wall structures (Fig. 4B–D). On the other hand, in the specimens with immunolabeling of different mAbs, a particular wall structure of a vessel element had different densities of bright particles, thereby showing differences in the kinds and/or abundance of the target pectic and hemicellulosic polysaccharides in the given wall structure (Fig. 4E, F). All of these have demonstrated that this technique can clarify the components and spatial distribution of polysaccharides in the lateral wall of vessel elements.

Xylem fiber cells in the grape stems had a thick secondary wall and occurred abundantly, forming a major mass in the secondary xylem (Fig. 1A). The secondary wall covered the whole lateral wall of a fiber cell except some regions containing simple interfiber pits where two adjacent fibers were separated by interfiber PMs (Figs 1A and 5). When a fiber cell was viewed in a longitudinal section, its lateral wall either had the intact secondary wall in place or revealed some secondary wall underlayers and even the primary wall layer due to removal of more superficial secondary wall layers (Fig. 5A–C). In the three negative controls for each immunogold labeling, bright particles were not or rarely observed on the interfiber PMs, intact secondary wall surface, secondary wall underlayers and exposed primary cell wall (Fig. 5A). In contrast, in the immunogold labeling tests, bright particles occurred with different densities on these wall structures depending on the mAb used (Fig. 5B–D). Different densities indicated quantitative differences of a specific polysaccharide or polysaccharide group in these structures.

Axial parenchyma cells occurred in a very small quantity and were only adjacent to vessels, while ray parenchyma cells formed multiseriate rays, some of which were adjacent to vessels (Fig. 1). Both types of xylem parenchyma cells had a relatively thin secondary wall and round or oval bordered PPs between adjacent cells of each kind (Fig. 6A, B). Each pit had a narrow secondary wall border, revealing a majority of its PM. In the wall regions without pits, removal of the more superficial secondary wall layer(s) may expose its underlayers and even the primary cell wall (Fig. 6A, B). In any of the three negative controls for each mAb tested, no or very few random bright particles were seen on any of the wall structural surfaces (Fig. 6A). In the specimens with immunogold labeling, bright particles were observed across a cell wall’s transverse section (Fig. 6B, C), different secondary wall underlayers and/or the primary cell wall layer (Fig. 6B, D), depending on the mAb used. The intact secondary wall surface did not have bright particles associated in the tests with all the mAbs used (Fig. 6B, C). These demonstrated the effectiveness of this technique in visualizing polysaccharides in the cell wall structures of xylem parenchyma.

Tyloses appeared as expanding, more or less spherical cellular structures in a vessel lumen as they developed from some parenchyma cells adjacent to the vessel (Figs 1A and 7A). Tyloses secreted a cell wall outside their protoplast and those of similar sizes in the vicinity were close in their developmental stage. Developmental details of tyloses were described in Sun *et al.* (2006, 2008). Tyloses in the three negative immunolabeling controls contained no or almost no bright particles on their wall surface, but displayed bright particles of different densities in the immunogold labeled specimens, depending on the cell wall mAb used for polysaccharide detection (Fig. 7B, C). The bright particles on tylose wall surface were more or less randomly distributed (Fig. 7D). These demonstrated that this technique can detect different pectic and hemicellulosic polysaccharides in tylose cell wall.

### Using immunogold SEM to reveal polysaccharide organization in the cell walls of xylem elements

The distribution and quantity of a polysaccharide in the different layers of a cell wall reflects the dynamics of its production during the cell wall formation. The polysaccharide components in the cell wall at a cell’s specific developmental stage provide an important clue for analysing metabolic activities and function of the cell wall and even the cell itself at that stage. The current immunogold SEM was applied to explore questions of these two aspects.

**Fig. 8.**
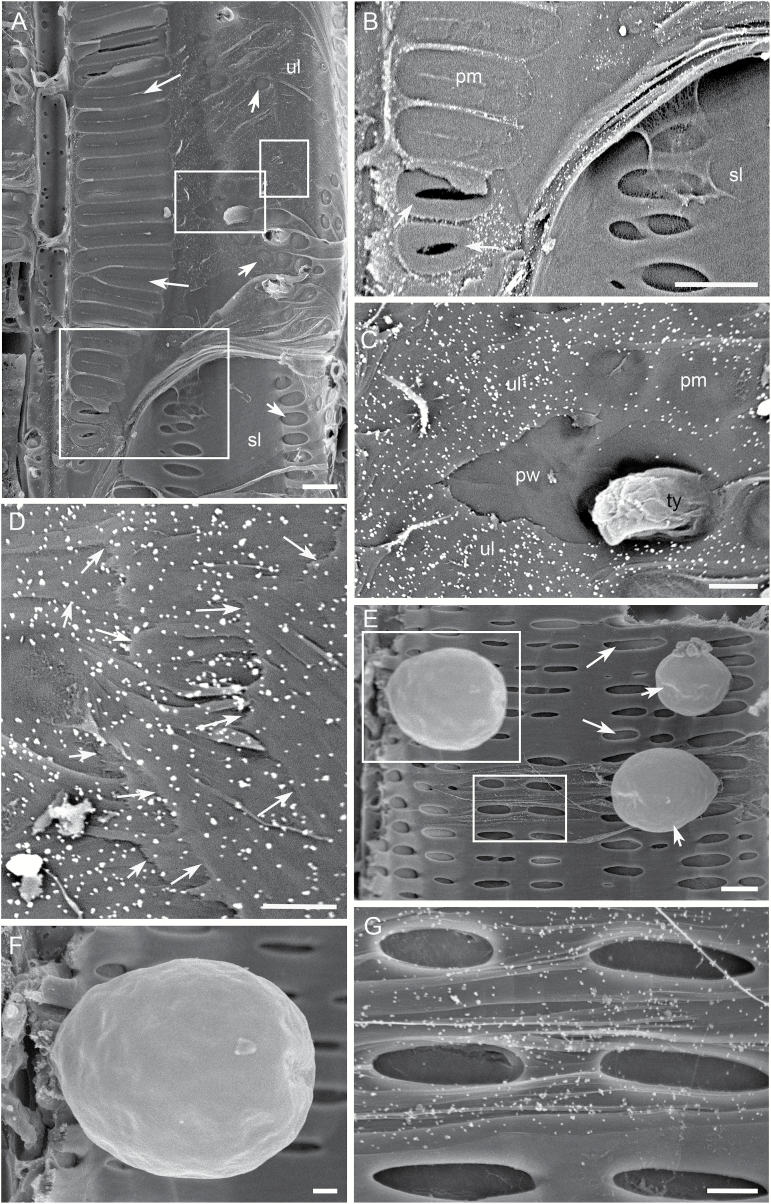
Representative images demonstrating detection of xylans in the cell wall of xylem elements from *V. vinifera* var. Chardonnay. The secondary xylem tissue was incubated with CCRC-M140 followed by the colloidal gold-conjugated goat anti-mouse IgG, and bright particles indicate the presence of xylans in the cell wall layers. (A) Tangential longitudinal surface of xylem tissue. A transected vessel has a direct wall contact with another vessel and axial parenchyma cells underneath through scalariform intervessel PMs (long arrows) and oval vessel-axial parenchyma PMs (short arrows), respectively. The vessel’s lateral wall has a small region with intact secondary wall (i.e. the superficial secondary wall layer (sl) in place) and a large region with some exposed PMs (arrows) and cell wall underlayers (ul) after removal of the superficial secondary wall layers. (B) Enlargement of the bottom frame in (A), showing one lateral wall region with the superficial secondary wall layer (sl) in place and another wall region with exposed intervessel PMs (pm). Several PMs are removed to reveal the free PM-facing secondary wall surface (arrows) of several pit borders of the vessel beneath. No bright particles are present on the intervessel PMs and the bottom and top free surfaces of the secondary cell wall, indicating the absence of xylans in these structures. (C) Enlargement of the middle frame in (A), showing several vessel-parenchyma PMs (pm) and the originally concealed primary cell wall region (pw) and a secondary wall underlayer (ul) right on the primary wall after removal of the superficial secondary wall layers. Bright particles are visible on the secondary wall underlayer but not on the primary cell wall and the vessel-parenchyma PMs, indicating the presence of xylans in the secondary wall underlayer but their absence in the primary wall and PMs. ty: tylose. (D) Enlargement of the top frame in (A), showing some secondary wall underlayers after removal of the superficial secondary wall layers. Arrows of a same length indicate the approximate location of one wall layer going under another with a more superficial layer on the right side of the arrows. Bright particles are present on all of the secondary wall underlayers revealed, indicating the constant presence of xylans throughout these wall layers. (E–G) Differential densities of bright particles in the cell walls of a vessel element and several tyloses. (E) A transected vessel showing vessel-parenchyma pits (long arrows) and developing tyloses (short arrows). (F) Enlargement of the top frame in (E). An expanding tylose does not have bright particles indicating the absence of xylans in its wall. (G) Enlargement of the bottom frame in (E), indicating the presence of xylans in the vessel’s secondary wall underlayers. Bar equals 10 µm in (A, B, E), and 3 µm in (C, D, F, G).

As an example of analysing the spatial distribution of polysaccharides in a cell wall, we used this immunogold SEM to reveal the spatial distribution and relative quantity of xylans in the lateral cell wall of vessel elements (Fig. 8A–D). The immunogold labeling with CCRC-M140 indicated that xylans displayed differential distributions in the different lateral wall layers of a vessel element. Xylans were not detected in primary cell wall, including intervessel PMs, vessel-parenchyma PMs and the primary cell wall portions which secondary wall layers are laid down on (Fig. 8A–C). This uniform absence of xylans suggests that the polysaccharides are not added to the primary cell wall during the differentiation of vessel elements. Xylans were also absent over the outermost surface (i.e. the pit border surface facing the PM) and innermost surface of intact secondary cell walls (Fig. 8A, B), but occurred with a constant quantity throughout all of the underlayers of the secondary cell wall (Fig. 8A, C, D). This indicates that the cell does not secrete xylans at the beginning and toward the end of secondary cell wall formation during the vessel element differentiation, but adds the xylans constantly to the secondary cell wall during the time in between.

Immunogold SEM can also reveal wall polysaccharide composition of xylem cells at specific developmental stages. As an example, we used this technique to analyse polysaccharide components in the cell wall of developing tyloses. As mentioned above, tyloses of similar sizes are at a similar developmental stage. When they are treated with different cell wall mAbs, their cell wall polysaccharide components can be revealed. Using CCRC-M1, JIM 5, and CCRC-M140, respectively, we found that tyloses at an early developmental stage contained abundant fucosylated XyGs (Fig. 7C, D), a small amount of weakly Me-HGs (Fig. 7B) and no xylans (Fig. 8E–G), indicating that a tylose secretes the differential quantities of these polysaccharides to its cell wall at this early stage.

## Discussion

Understanding the polysaccharide composition and organization in a cell wall is essential to reveal cell wall structure and function (Cosgrove, 2005; Albersheim *et al.*, 2010; Keegstra, 2010). An expanding number of molecular probes generated for detecting cell wall polysaccharides have greatly facilitated this kind of investigation (Willats *et al.*, 2001*b*; Knox, 2008; Pattathil *et al.*, 2010). In this study, we have explored an immunogold SEM technique for visualizing polysaccharides in the cell walls of diverse xylem elements. After a variety of tests and adjustments to the protocol, the technique has proven equally reliable and effective for detecting cell wall pectic and hemicellulosic polysaccharides in vessel elements, fibers, axial and ray parenchyma cells, and tyloses. As demonstrated in this study, this technique, along with the increase in resolution and field of view of SEM and the growing number of molecular probes for cell wall polysaccharides, can provide significant insight into polysaccharide architecture and function of cell walls.

This immunogold SEM technique has clear advantages over the two other *in situ* cell wall polysaccharide analysis techniques: IFM and immunogold TEM. These two techniques were originally developed to localize polysaccharides on the transverse section of a cell wall but they can gain information at different structural levels. IFM is useful in detecting cell wall polysaccharides at the cellular or tissue level. However, the inherent limitation in resolution as a light microscopy technique makes it difficult for IFM to reveal either spatial differences of polysaccharides throughout the thickness of a cell wall or structural details of the cell wall investigated. Therefore, IFM is difficult for a simultaneous analysis of polysaccharide composition and cell wall structure (Sun *et al.*, 2011). On the other hand, immunogold TEM is suitable for analysing polysaccharide composition across the thickness of a cell wall. The technique involves the use of a secondary Ab conjugated with colloidal gold nanoparticles (e.g. 10 or 15 nm) for target molecule detection (Herman, 1988, 2000; Ferguson *et al.*, 1998; Murtey, 2016). By directly localizing the distribution and quantity of the gold nanoparticles at high magnifications, this technique can reveal the spatial distribution and richness of specific polysaccharides across the cell wall thickness. When combined with the cell wall structural details revealed also at high magnifications, the information gained is helpful for a delicate analysis of cell wall structure and polysaccharide composition. However, one major disadvantage of this technique is that polysaccharide detection is made mostly within a restricted wall area and this therefore hinders a more comprehensive understanding of polysaccharide composition at the tissue level (Liners and Van Custem, 1992; Roy *et al.*, 1994; Plavcová and Hacke, 2011; Herbette *et al.*, 2015).

In analysing polysaccharides in the transverse section of a cell wall, the immunogold SEM technique has combined the main advantages of the two techniques discussed above while negating their disadvantages. In the immunogold SEM, there is practically no limitation on specimen size, so analysis of cell walls of different cells or cell types in a large tissue area is possible. This provides obvious benefits over studying different structures with multiple smaller specimens. As indicated in ‘Materials and methods’, the immunocytochemical method involves a complicated procedure including incubations in multiple Abs and silver enhancement treatment, which may be affected by chemical concentrations, treatment time, and/or temperature. Using a large specimen may avoid any potential errors derived from subtle differences that likely occur to multiple smaller specimens processed separately, contributing to achieve accurate qualitative and quantitative analyses on the cell wall polysaccharide architecture of diverse cell types. On the other hand, like the immunogold TEM, the immunogold SEM technique is capable of pinpointing a cell wall or wall portion of any xylem element for a delicate polysaccharide composition and structure analysis at high resolution. Therefore, this immunogold SEM technique is valuable for exploring polysaccharide composition and distribution in the transverse section of cell walls at a tissue level, a very restricted wall region, and any level in between, just as demonstrated in this study.

The immunogold SEM protocol developed in this study is also powerful in detecting polysaccharides in different layers of a cell wall. This feature even generates some incomparable advantages over either of the other two techniques, especially in analysing the cell wall polysaccharide architecture of xylem elements. As shown in this study, SEM specimens exposing a tangential or radial longitudinal surface of a xylem tissue clearly displayed the lateral walls of the different xylem cells. With this immunogold SEM protocol, the entire exposed lateral wall of each type of xylem cell can be viewed at once with SEM, allowing for a simultaneous analysis on the polysaccharide composition and wall structure across its entire surface up to high resolution (Figs 4–8). IFM was also used in some cases to explore certain polysaccharides across a cell wall surface, leading to some interesting findings (Casero and Knox, 1995; Willats *et al.*, 2001*a*; Ordaz-Ortiz *et al.*, 2009; Sun *et al.*, 2011). However, the limited resolution of light microscopy restricted this simultaneous high-resolution analysis of polysaccharides and wall structure. In addition, trimming a xylem sample for the immunogold SEM may cause removal of the superficial secondary wall layer(s) in some wall regions of a lateral cell wall, exposing different secondary wall underlayers and even the primary cell wall. As demonstrated in this study, the immunogold SEM technique can be used to clearly visualize polysaccharide components in these exposed underlayers (Fig. 8). Since these underlayers represent the chronological order of wall deposition, revealing their polysaccharide compositions can not only clarify the spatial distribution and quantity of polysaccharides in the cell wall, but also provide insight into the metabolism and function of the cell wall and even the cell itself.

In addition to the polysaccharide analysis in different wall layers, the immunogold SEM is also an excellent tool to explore any potential differences in the composition and quantity of polysaccharides among different structures in the same lateral wall of a xylem cell. Intervessel PMs, vessel-parenchyma PMs and wall regions with the intact secondary cell wall may all be found in the same lateral wall of a vessel element but exhibit some important functional differences (Esau, 1977; Evert, 2006). Similar situations also occur with other xylem elements. With the immunogold SEM technique, the current study has revealed that certain groups of polysaccharides were present in large quantities in one wall structure, but were absent or occurred in small amounts in other wall structures (Figs 4–8). Information gained from this aspect is vital to understand the chemical compositional basis that underlines the structural and functional differentiations of these wall features.

PMs separating two adjacent xylem cells are exposed to the two cells’ lumens. They are also subjected to chemical modifications, resulting in some structural and polysaccharide compositional changes that consequently better fit or alter their functions (Esau, 1977; Flincher and Stone, 1981; Tyree and Zimmermann, 2002). It has been suggested that cell autolysis during the maturation of a vessel element causes removal of some polysaccharides from the PM regions (Meylan and Butterfield, 1972; O’Brien, 1974; Sperry *et al.*, 1991; van Doorn *et al.*, 2011). Chemical modifications on the vessel-parenchyma PMs should also take place, so the PMs can be involved in tylose formation and/or gel secretion (Evert, 2006; Sun *et al.*, 2006, 2007, 2008). Some polysaccharides in intervessel PMs are believed to interact with the solute ions conducted in the vessels to change the porosity of the PMs, so they may be involved in regulating the water transport through the vessel system (Zwieniecki *et al.*, 2001; Lopez-Portillo *et al.*, 2005; Wheeler *et al.*, 2005; van Ieperen, 2007). However, details on these PM modifications, etc. mostly remain unknown. As demonstrated in this study, our immunogold SEM protocol can detect the presence and richness of polysaccharides in both intervessel and vessel-parenchyma PMs. Therefore, we believe that this technique will be certainly beneficial to explore any potential dynamic polysaccharide changes during these diverse forms of PM modifications in xylem cells.

Most devastating plant vascular diseases of commercial importance are caused by pathogens that inhabit the vessel system of host plants (Beckman, 1987; Hopkins, 1989; Barras *et al.*, 1994; Bove and Garnier, 2002). This immunogold SEM technique can be used to analyse pathogen–host plant interactions in these vascular diseases. Symptom development of a vascular disease is largely dependent upon its pathogen’s modifications on intervessel PMs and possibly also on vessel-parenchyma PMs of a host plant. On one hand, modifications of intervessel PMs help the pathogen to achieve a systemic spread throughout the vessel system that is needed for symptom development (Purcell and Hopkins, 1996; Newman *et al.*, 2003, 2004; Ellis *et al.*, 2010; Sun *et al.*, 2011, 2013). On the other hand, modifications of vessel-parenchyma PMs may be related to the formation of tyloses and gels that occlude the vessel lumens, thereby affecting the pathogen’s spread and/or the host plant’s water transport (Pearce, 1991; Rioux *et al.*, 1995; Clérivet *et al.*, 2000; Sun *et al.*, 2006, 2013). Many of these pathogen-induced modifications of intervessel PMs are thought to be caused by the pathogen’s cell wall degrading enzymes (CWDEs) that may remove certain polysaccharides from the PMs, increasing their porosity for the passage of the pathogen (Roper *et al.*, 2007; Pérez-Donoso *et al.*, 2010; Sun *et al.*, 2011; Kubicek *et al.*, 2014). However, details of the CWDEs and their target cell wall polysaccharides are still lacking. In some grapevines infected by the bacterial pathogen *Xylella fastidiosa*, we have observed a whole degradation process of intervessel PMs in some pathogen-dwelling vessels, suggesting the removal of certain polysaccharides from the intervessel PMs during the degradation process (Sun *et al.*, 2011, 2013). We are applying this immunogold SEM technique to identify the target PM polysaccharides of the pathogen CWDEs and the PM modification/degradation dynamics in relation to the disease symptom development. Such information has an obvious value in better understanding pathogen–host plant interactions and further revealing vascular disease susceptibility mechanism of host plants.
